# Adaptative biochemical pathways and regulatory networks in *Klebsiella oxytoca* BAS-10 producing a biotechnologically relevant exopolysaccharide during Fe(III)-citrate fermentation

**DOI:** 10.1186/1475-2859-11-152

**Published:** 2012-11-23

**Authors:** Giuseppe Gallo, Franco Baldi, Giovanni Renzone, Michele Gallo, Antonio Cordaro, Andrea Scaloni, Anna Maria Puglia

**Affiliations:** 1 Dipartimento di Scienze e Tecnologie Molecolari e Biomolecolari (STEMBIO), Università di Palermo Viale delle Scienze, ed. 16, Parco d'Orleans II, Palermo, 90128, Italy; 2 Dipartimento di Scienze Molecolari e Nanosistemi (DSMN), Cà Foscari, Università di Venezia, Calle Larga S. Marta, Dorsoduro 2137, Venezia, 30123, Italy; 3 Laboratorio di Proteomica e Spettrometria di Massa, ISPAAM, Consiglio Nazionale delle Ricerche, Naples, 80147, Italy

## Abstract

**Background:**

A bacterial strain previously isolated from pyrite mine drainage and named BAS-10 was tentatively identified as *Klebsiella oxytoca*. Unlikely other enterobacteria, BAS-10 is able to grow on Fe(III)-citrate as sole carbon and energy source, yielding acetic acid and CO_2_ coupled with Fe(III) reduction to Fe(II) and showing unusual physiological characteristics. In fact, under this growth condition, BAS-10 produces an exopolysaccharide (EPS) having a high rhamnose content and metal-binding properties, whose biotechnological applications were proven as very relevant.

**Results:**

Further phylogenetic analysis, based on 16S rDNA sequence, definitively confirmed that BAS-10 belongs to *K. oxytoca* species. In order to rationalize the biochemical peculiarities of this unusual enterobacteriun, combined 2D-Differential Gel Electrophoresis (2D-DIGE) analysis and mass spectrometry procedures were used to investigate its proteomic changes: i) under aerobic or anaerobic cultivation with Fe(III)-citrate as sole carbon source; ii) under anaerobic cultivations using Na(I)-citrate or Fe(III)-citrate as sole carbon source. Combining data from these differential studies peculiar levels of outer membrane proteins, key regulatory factors of carbon and nitrogen metabolism and enzymes involved in TCA cycle and sugar biosynthesis or required for citrate fermentation and stress response during anaerobic growth on Fe(III)-citrate were revealed. The protein differential regulation seems to ensure efficient cell growth coupled with EPS production by adapting metabolic and biochemical processes in order to face iron toxicity and to optimize energy production.

**Conclusion:**

Differential proteomics provided insights on the molecular mechanisms necessary for anaeorobic utilization of Fe(III)-citrate in a biotechnologically promising enterobacteriun, also revealing genes that can be targeted for the rational design of high-yielding EPS producer strains.

## Background

Several species of enterobacteria use citrate as sole carbon and energy source. This capability is firstly due to appropriate transporters for citrate up-take, such as the citrate-specific proteins CitH and CitS [1,2] or like the tripartite tricarboxylate transporter (TTT) TctABC system able to transport several tricarboxylic acids into the bacterial cell [3] or like the ferric citrate transport system (the product of the *fecABCDE* operon) that shuttles the Fe(III)-citrate complex into the cytoplasm [4-7].

During aerobiosis, intracellular citrate is catabolized throughout the TCA cycle. Under anaerobic conditions, when TCA cycle is down-regulated, enterobacteria species, like *Klebsiella pneumoniae* and *Salmonella typhimurium*, can grow on citrate by a Na(I)-dependent pathway, forming acetic acid and CO_2_ as final metabolites [1,8,9]. Genes specific for anaerobic citrate fermentation, such as those coding for regulators (*citAB*), catabolic enzymes (*citCDEFG* and *oadGAB*) and citrate transporters (*citS* and *citW*), have been identified in these bacteria [1,2,8,10]. The presence of sodium is essential for citrate symport by CitS and for the activity of *oadGAB* gene products that form the oxaloacetate decarboxylase complex. In fact, oxaloacetate decarboxylase converts oxaloacetate into pyruvate and pumps sodium externally to synthesize ATP [1,2,8,9].

Generally, iron is one of the major limiting nutrients [11] and citrate-fermenting enterobacteria do not usually thrive on high concentrations of Fe(III)-citrate as sole carbon and energy source [12]. Indeed, there are habitats where the abundance of Fe(III) is so high, like in pyrite mine drainages, which represents one of the major elements to make rust-red the acidic waters. In this case, iron can represent an environmental hazard for life, especially for its oxidative properties and for the presence of other metals which increase the total toxicity of mine drainages and cause a significant reduction of microbial biodiversity [13-15]. Only specialized species can survive in extreme habitats with high heavy metal concentrations and carbon-depleted conditions and *Enterobacteraceae* are not expected to survive in such environments. Nevertheless, an enterobacterial strain was isolated under an iron mat formed by waters leached from pyrite mine drainages of Colline Metallifere, Tuscany, Italy [16]. This isolate, named BAS-10, was tentatively identified as *K. oxytoca* on the basis of partial (422 nt) 16S rDNA sequence and API Enterotube test [16]. Unique among *Klebsiella* strains, BAS-10 can ferment and proliferate on Fe(III)-citrate as sole carbon and energy source, forming acetic acid and CO_2_ coupled with Fe(III) reduction to Fe(II) [12]. Under these growth conditions, BAS-10 produces an EPS made of rhamnose (57.1%), glucuronic acid (28.6%) and galactose (14.3%), which shows metal-binding properties [17,18]. Although extracellular EPS have been reported over recent decades and their composition, structure, biosynthesis and functional properties have been extensively studied, only a few have been industrially developed [19].

Chelating and sugar compositional properties of BAS-10 EPS are of high interest for potential biomedical, food, and environmental applications [18-20]. Further studies on BAS-10 physiology may be useful to develop efficient fermentation processes for EPS production. In order to investigate iron-dependent biochemical and metabolic adaptations and regulatory networks thereof during anaerobic growth on Fe(III)-citrate as the sole carbon source, a differential proteomic approach was used. In particular, proteomic repertoires from BAS-10 grown on Fe(III)-citrate under anaerobiosis, on Na-citrate under anaerobiosis and on Fe(III)-citrate under aerobiosis were comparatively evaluated.

## Results and discussion

### Phylogenetic identification

**Figure 1:**
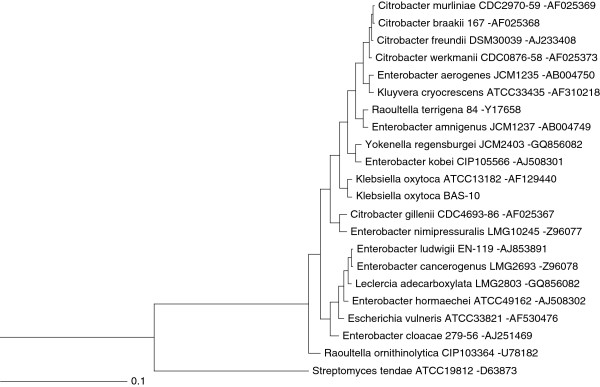
**Phylogenetic analysis of BAS-10 strain performed by using 16S rDNA sequences.** The first twenty hits from BLAST analysis performed by selecting only cultivable reference strains were used to generate the phylogenetic three. The 16S rDNA sequence of *Streptomyces tendae* was used as outgroup. Distance unit is based on sequence identity. NCBI accession number of each 16S rDNA is reported after hyphen.

Physiological studies [12], EPS composition and metal-binding activity thereof [17,18] revealed characteristic peculiarities of BAS-10 strain. Thus, a sequence of 1447 nt gene was generated from BAS-10 16S rDNA (Additional File 1) to perform phylogenetic clustering. Two ClustalW analyses were performed by using the first twenty hits from Blast analysis, selecting only cultivable reference or whole strains from NCBI database (http://blast.ncbi.nlm.nih.gov/) [21], respectively. Phylogenetic threes revealed that BAS-10 is related to *K. oxytoca* reference strain (Figure 1) and can be clustered into a group comprising other five very close related strains classified as *K. oxytoca* (Additional file 1 Figure S1). This investigation definitively confirmed previous taxonomical observations on BAS-10 [16].

### Proteomic analysis

To investigate iron-dependent cell processes and regulatory networks thereof associated to citrate fermentation and EPS production, BAS-10 was cultivated: i) under aerobic conditions on Fe(III)-citrate (FEC) as sole carbon source; ii) under anaerobic conditions using FEC or iii) Na(I)-citrate (NAC) as sole carbon source. Then, bacterial proteomes were analyzed using anaerobic FEC growth as pivotal condition. In particular, the “anaerobic FEC Vs aerobic FEC” differential analysis was carried out to reveal proteins that are required for anaerobic growth on FEC while “anaerobic FEC Vs anaerobic NAC” differential analysis was carried out to reveal proteins whose abundance is iron-dependent during anaerobic growth. In all cases, BAS-10 biomass samples were all harvested at late exponential growth phases, when anaerobic growth on FEC is coupled with iron-binding EPS production [12,16] at an average yield of 6.7 g/L (dry weight EPS *per* culture volume). Proteomic 2D-DIGE maps showed the occurrence of 869 reproducible protein spots (i. e. detected in all 2D-Gels) which were used to create the match set for comparative analysis. According to the criteria reported in the experimental section for identification of deregulated proteins, comparative analysis between FEC medium cultivations under anaerobic or aerobic conditions revealed 27 and 32 protein spots up- and down-regulated under anaerobic conditions, respectively (Table 1; Additional file 1 Figure S2). On the other hand, comparative analysis between cultivations in FEC and NAC media under anaerobic conditions revealed 28 and 42 protein spots as up- and down-regulated in the presence of Fe(III) (Table 1; Additional file 1 Figure S2). Among these protein spots, 46 resulted differentially abundant with a concordant profile in both analyses (anaerobic FEC *vs* aerobic FEC and anaerobic FEC *vs* anaerobic NAC) (Table 1), thus indicating that these proteins are specifically associated with anaerobic growth on FEC. The selected differentially abundant protein spots (83 in number) were alkylated, trypsinolyzed and identified by MALDI-TOF-peptide mass fingerprinting (PMF) and nanoLC-ESI-LIT-MS/MS procedures (Table 1 and Additional file 1 Table S1). Corresponding protein entries were clustered into functional groups, according to KEGG (http://www.genome.ad.jp/kegg/kegg2.html) [22], EcoCyc (http://ecocyc.org/) [23] and ExPASy (http://www.expasy.org) [24] databases (Additional file 1 Figure S3). Among the identified proteins, the most represented ones were related to central carbon metabolism -including glycolysis/gluconeogenesis, tricarboxylic acid (TCA) cycle, citrate fermentation, pentose phosphate (PP) pathway -and membrane transport (Table 1; Additional file 1 Figure S3).

**Table 1:** **Functional classification and relative fold change of *K. oxytoca *BAS-10 proteins differentially expressed during anaerobic growth in FEC**

**Spot**	**Protein name**	**Acronym**	**NCBI code**	**Theor. pI/Mr (kDa)**	**Exp. pI/Mr (kDa)**	**Functional class**^**a**^	**Relative fold change**^**b**^**(−O**_**2**_**FEC Vs +O**_**2**_**FEC)**	**Relative fold change**^**b**^**(−O**_**2**_**FEC Vs -O**_**2**_**NAC)**
1	NADP-specific glutamate dehydrogenase	GLDH	376386651	6.73/49	6.26/45	Amino Acid Metabolism	1	2.6
5^*^	Nitrogen regulatory protein P-II 2	GlnK	376394829	5.84/12	5.62/13	Amino Acid Metabolism	−3.2	−8.7
6	Nitrogen regulatory protein P-II 2	GlnK	376394829	5.84/12	5.62/13	Amino Acid Metabolism	1	−2.7
7	Nitrogen regulatory protein P-II 2	GlnK	376394829	5.84/12	5.20/13	Amino Acid Metabolism	1	−2.2
3^*^	Ketol-acid reductoisomerase	IlvC	376396684	5.25/54	5.04/54	Amino Acid Metabolism	2.3	2.2
4	D-3-phosphoglycerate dehydrogenase	PHGDH	365906793	6.06/44	5.92/46	Amino Acid Metabolism	1	−2.7
8	Major outer membrane lipoprotein 1	Lpp	376401994	9.36/8	4.16/12	Cell wall	1	3.4
9	Major outer membrane lipoprotein 1	Lpp	376401995	9.36/9	4.04/12	Cell wall	1	3.4
10	Major outer membrane lipoprotein 1	Lpp	376401994	9.36/8	4.44/12	Cell wall	1	1.7
37	Aerobic respiration control protein ArcA	ARCA	376394332	5.30/27	5.04/29	Central carbon metabolism	1.9	1
19^*^	Glucose-specific phosphotransferase enzyme IIA component	EIIA^Glc^	376399349	4.73/18	4.6/22	Central carbon metabolism	−1.9	−1.7
20^*^	Glucose-specific phosphotransferase enzyme IIA component	EIIA^Glc^	376399349	4.73/18	4.62/20	Central carbon metabolism	−1.5	−1.4
17^*^	Hypothetical protein HMPREF9694_04828 (dihydrolipoyllysine-residue succinyltransferase, E2 component)	SucB	376395176	5.74/44	5.39/54	Central carbon metabolism	−2.8	−3.6
41	Succinyl-CoA synthetase subunit alpha	A-SCS	365909192	5.89/30	5.65/30	Central carbon metabolism	−3.9	1
33	Succinyl-CoA synthetase subunit beta	B-SCS	365909191	5.35/42	5.05/43	Central carbon metabolism	−1.8	1
42	Fumarate hydratase class II	FH	376401593	6.02/50	5.92/46	Central carbon metabolism	1	−2.7
2^*^	Fumarate reductase flavoprotein subunit	FrdA	376396036	5.60/66	5.44/77	Central carbon metabolism	2.1	2.6
22^*^	Malate dehydrogenase	MDH	376398117	5.57/33	5.34/34	Central carbon metabolism	−3.1	−2.7
23^*^	Malate dehydrogenase	MDH	376398117	5.57/33	5.08/29	Central carbon metabolism	−1.9	−2.7
14	Citrate lyase beta subunit	CitE	376376734	5.33/31	4.87/29	Central carbon metabolism	1.9	1
26	Citrate lyase beta subunit	CitE	376376734	5.33/31	5.07/31	Central carbon metabolism	4.9	1
36^*^	Citrate lyase beta subunit	CitE	376376734	5.33/31	5.19/29	Central carbon metabolism	3.0	2.4
15^*^	Citrate lyase, alpha subunit	CitF	365908401	5.94/55	5.98/55	Central carbon metabolism	8.7	9.0
16^*^	Citrate lyase, alpha subunit	CitF	365908401	5.94/55	5.89/55	Central carbon metabolism	14.1	11
24^*^	Oxaloacetate decarboxylase alpha chain	OadA	376376739	5.44/63	5.04/64	Central carbon metabolism	10.4	8.7
25^*^	Oxaloacetate decarboxylase alpha chain	OadA	376376739	5.44/63	4.7/43	Central carbon metabolism	3.7	2.8
81^*^	Oxaloacetate decarboxylase alpha chain	OadA	376376739	5.44/63	4.74/43	Central carbon metabolism	4.0	2.4
29^*^	Pyruvate formate lyase	PFL	376393708	5.63/85	5.09/80	Central carbon metabolism	2.7	12.4
30^*^	Pyruvate formate lyase	PFL	376393708	5.63/85	5.3/80	Central carbon metabolism	2.1	15.4
31^*^	Pyruvate formate lyase	PFL	376393708	5.63/85	5.37/80	Central carbon metabolism	2.9	12.1
32^*^	Pyruvate formate lyase	PFL	376393708	5.63/85	5.44/77	Central carbon metabolism	2.7	12.4
35^*^	Pyruvate formate lyase	PFL	376393708	5.63/85	5.16/43	Central carbon metabolism	2.3	3.4
27^*^	Phosphotrans-acetylase	PTA	365911544	5.26/77	5.27/65	Central carbon metabolism	3.6	2.7
12^*^	Acetate kinase A/propionate kinase 2	ACK	365911543	5.84/44	5.39/27	Central carbon metabolism	4.1	1.7
46^*^	Triosephosphate isomerase	TIM	376397984	5.82/26	5.5/26	Central carbon metabolism	1.6	1.5
38^*^	Pyruvate kinase	PK	365911037	6.00/52	5.78/55	Central carbon metabolism	1.9	1.9
21^*^	Glyceraldehyde-3-phosphate dehydrogenase	GAPDH	376401359	6.33/36	6.2/31	Central carbon metabolism	2.2	1.5
28	Pyruvate dehydrogenase subunit E1	PDH	365908482	5.47/100	5.52/67	Central carbon metabolism	2.4	1
40	3-Methyl-2-oxobutanoate hydroxymethyltransferase	PanB	365908516	5.64/28	5.34/29	Cofactor biosynthesis	1	−2.5
49	6,7-Dimethyl-8-ribityllumazine synthase	RibH	365908789	5.12/16	4.87/14	Cofactor biosynthesis	1	−2
51	DNA protection during starvation protein	DPS	376393609	5.72/19	5.61/16	DNA ricombination, replication and repair	−1.5	1
50	DNA-binding protein HU-alpha	HU-2	376396790	9.40/9	9.07/11	DNA ricombination, replication and repair	1	−2.3
52^*^	ATP synthase subunit beta	ATPβ	376397178	4.93/50	4.81/51	Energy metabolism	2.5	2.2
53^*^	ABC transporter arginine-binding protein 1	ArtJ	376393664	6.90/27	5.98/25	Membrane Transport	4.9	7.4
57^*^	Glutamate and aspartate transporter subunit	DEBP	365909131	8.61/33	7.85/30	Membrane Transport	−7.7	−6.7
58^*^	Glutamine-binding periplasmic protein	GlnH	376388520	8.74/27	5.81/26	Membrane Transport	−1.9	−2.4
59^*^	Glutamine-binding periplasmic protein	GlnH	376388520	8.74/27	7.8/25	Membrane Transport	−3.1	−6.2
60^*^	Glutamine-binding periplasmic protein	GlnH	376388520	8.74/27	6.87/25	Membrane Transport	−2.3	−3.8
34^*^	Leucine ABC transporter subunit substrate-binding protein LivK	LivK	365907255	5.71/40	5.03/42	Membrane Transport	3.0	3.5
61^*^	Maltose ABC transporter periplasmic protein	MBP	365907919	6.88/43	5.96/40	Membrane Transport	−7	−14
56^*^	D-galactose-binding periplasmic protein	MGLB	376381367	6.14/36	5.42/29	Membrane Transport	−2.6	−2.5
13	Outer membrane protein A	OmpA	376393752	5.98/38	4.98/31	Membrane Transport	2.7	1
39	Outer membrane protein A	OmpA	376393752	5.98/38	5.5/28	Membrane Transport	1	−1.6
43	Outer membrane protein A	OmpA	376393752	5.98/38	4.78/32	Membrane Transport	1	−1.5
44	Outer membrane protein A	OmpA	376393752	5.98/38	4.86/32	Membrane Transport	1	−1.6
62	Outer membrane protein A	OmpA	376393752	5.98/38	5.17/32	Membrane Transport	1	−2.3
63	Outer membrane protein A	OmpA	376393752	5.98/38	5.03/32	Membrane Transport	1	−3.1
64	Outer membrane protein A	OmpA	376393752	5.98/38	5.39/22	Membrane Transport	1	−5
88	Outer membrane protein A	OmpA	376393752	5.98/38	5.46/27	Membrane Transport	1	−3.7
45	Outer membrane protein W	OmpW	365909926	6.17/23	5.2/22	Membrane Transport	−4.0	1
18^*^	Hypothetical protein HMPREF9694_01670 (tricarboxylic transport)	TctC	376400424	8.61/35	6.7/26	Membrane Transport	−6.5	−5.0
48^*^	Hypothetical protein HMPREF9694_01670 (tricarboxylic transport)	TctC	376400424	8.61/35	7.8/29	Membrane Transport	−4.1	−8.0
71^*^	Hypothetical protein HMPREF9694_01670 (tricarboxylic transport)	TctC	376400424	8.61/35	8.37/29	Membrane Transport	−10	−6.2
65^*^	Carbamoyl phosphate synthase small subunit	CPSase	365908404	5.79/42	5.4/42	Nucleotide metabolism	−2	−3.3
66^*^	Multifunctional nucleoside diphosphate kinase	NdK	365911711	5.55/15	5.38/14	Nucleotide Metabolism	−2.6	−4.2
67^*^	Nucleoside diphosphate kinase	NdKs	376399440	5.55/15	5.42/14	Nucleotide Metabolism	−1.8	−4.1
89	Isochorismatase hydrolase		365909686	5.61/24	5.36/26	Other	−1.6	1
68	Adenosine-3'(2'),5'-bisphosphate nucleotidase	CysQ	365908080	5.67/28	5.31/29	Other	1	−2.1
72	Alkyl hydroperoxide reductase subunit C	AHPC	376395087	5.03/21	4.95/23	Oxido reduction	−2.3	1
76	Superoxide dismutase [Fe]	FeSOD	376402095	5.75/21	5.49/22	Oxido reduction	1	3.1
73	Superoxide dismutase [Mn]	MnSOD	376396893	6.23/23	5.84/25	Oxido reduction	1	−2.6
74^*^	Superoxide dismutase [Mn]	MnSOD	376396893	6.23/23	6.01/24	Oxido reduction	−2.8	−6.3
75^*^	Superoxide dismutase [Mn]	MnSOD	376396893	6.23/23	5.62/24	Oxido reduction	−2	−4.5
11	Thiol peroxidase	TPX	376401245	4.67/18	4.8/19	Oxido reduction	1	−1.9
79	Chaperonin	CHA10	376396016	5.38/10	4.97/16	Protein metabolism	1	−3.2
82^*^	Elongation factor G	EF-G	365907166	5.17/77	5.62/50	Protein metabolism	1.8	1.9
86^*^	Ribosomal protein L1	RPL1	376396770	9.56/25	7.77/27	Protein metabolism	−2	−2.2
87^*^	Ribosomal protein L1	RPL1	206564980	9.56/25	7.56/28	Protein metabolism	−1.7	−2.1
80	Ribosomal protein L13	RPL13	365907069	9.60/16	8.84/16	Protein metabolism	1	−3.9
85^*^	Peptidyl-prolyl cis-trans isomerase SurA	SurA	365908414	6.42/47	5.68/48	Protein metabolism	−2.1	−2.3
78	Autonomous glycyl radical cofactor	GrcA	365911773	4.82/14	4.71/12	Stress response	−1.9	1
69^*^	Osmotically-inducible protein Y	OSMY	376376350	8.67/21	5.84/22	Stress response	−3.0	−3.7
47	Universal stress protein F	USF	376401205	5.46/16	5.11/14	Stress response	−1.5	1

Fold change refers to normalized spot volumes as calculated by *in silico* analysis of 2D-DIGE protein maps. Positive and negative values stand for up- and down-regulation in anaerobic growth on FEC, respectively. Protein spots differentially abundant in anaerobic FEC with the respect to both aerobic FEC and anaerobic NAC are marked by an asterisk.

^a^Functional classification was performed according to KEGG database (http://www.genome.jp/kegg/).

^b^Relative fold change was measured according to the criteria reported in the experimental section. FEC: Fe(III)-citrate containing medium. NAC: Na(I)-citrate containing medium. +O_2_: aerobiosis. -O_2_: anaerobiosis.

**Figure 2:**
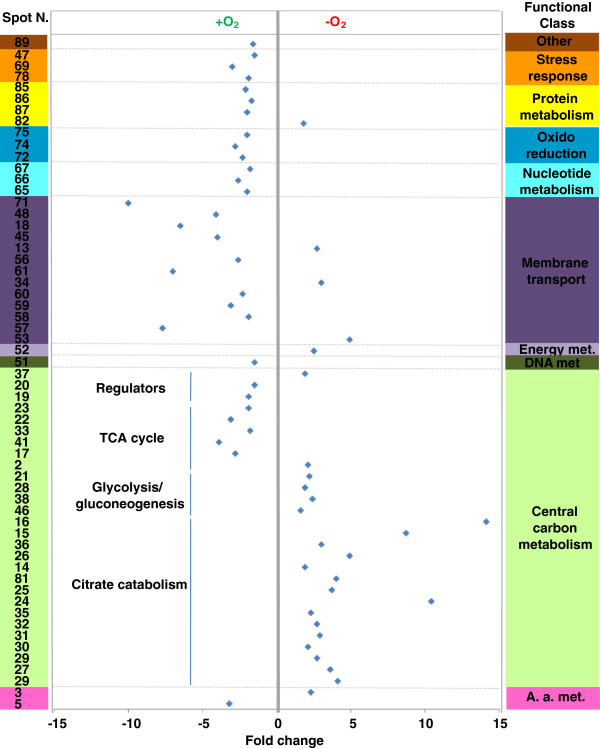
**Functional distribution and abundance fold change (blue diamond) of differentially represented protein spots resulting from the proteomic comparison between anaerobic and aerobic growth on FEC.** Fold change and spot number refer to Table 1. A. a. stands for Amino acid. Met. stands for metabolism.

### Proteins required for Fe(III)-citrate fermentation

**Figure 3:**
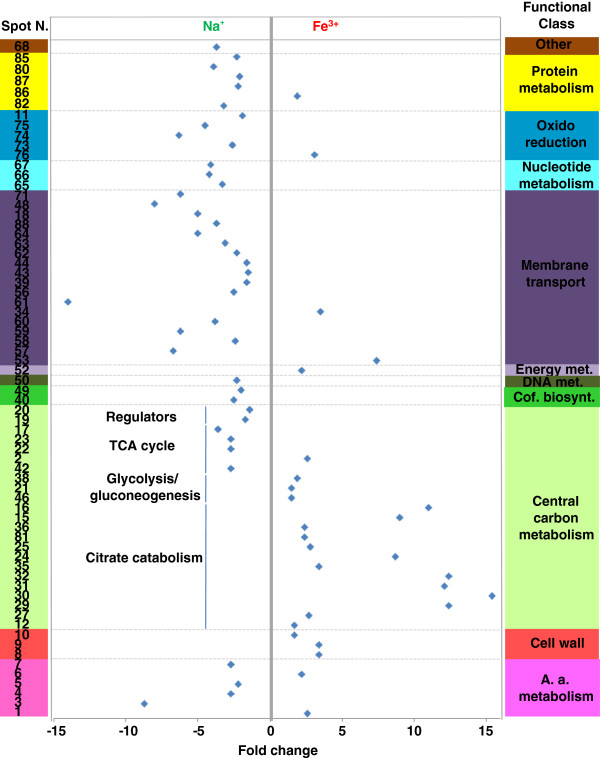
**Functional distribution and abundance fold change (blue diamond) of differentially represented protein spots resulting from the proteomic comparison between anaerobic growth on FEC and NAC.** Fold change and spot number refer to Table 1. A. a. stands for Amino acid. Met. stands for metabolism. Cof. biosynth. stands for Cofactor biosynthesis.

The differential proteomic analysis revealed up-regulation of citrate lyase α- and β-subunit (CitE and CitF), oxaloacetate decarboxylase α-subunit (OadA), pyruvate formate lyase (PFL), phosphotrans-acetylase (PAT) and acetate/propionate kinase (ACK) in anaerobic FEC with respect to both aerobic FEC and anaerobic NAC (Table 1; Figures 2 and 3). These proteins are known to be involved in anaerobic citrate fermentation, eventually converting citrate to acetate with production of ATP. In particular, CitF and CitE are part of the citrate lyase complex, the key enzyme in initiating the anaerobic utilization of citrate, which is responsible for catalyzing the conversion of citrate into acetate and oxaloacetate. The oxaloacetate decarboxylase complex, constituted by alpha, beta and gamma subunits, catalyzes the second step of citrate fermentation by converting oxaloacetate into pyruvate, the latter being the substrate of PFL, a typical enzyme of enterobacteria growing under anaerobic conditions [25], that generates formate and acetyl-CoA. Phosphotrans-acetylase (PAT) catalyzes conversion of acetyl-CoA into acetyl-P, which finally transfers the phosphate group to ADP to yield acetic acid and ATP in the ACK-catalyzed last step of citrate fermentation. Different forms of CitE, CitF, OadA and PFL were identified in *K. oxytoca* BAS-10 2D-protein maps (Table 1, Figures 2 and 3; Additional file 1 Table S1 and Figure S2). The occurrence of several protein forms having Mw and/or pI different from the predicted one would imply that protein activity could be controlled by post-translational modifications (PTMs), like covalently-bound charged small molecules (as in the case horizontal spot trains) or proteolytic digestion (as in the case of protein isoform with reduced Mw). Indeed, PTMs, like acetylation, succinylation and radical formation have already been shown for PFL activity regulation [26-28], thus giving count for the generation of different protein spots in 2D-maps. In the case of OadA, biotinylation in lysine, reported to be crucial for oxaloacetate decarboxylase activity [29], could explain the slight increase of the measured Mw while the generation of reduced Mw species is likely to be due to proteolytic fragmentation. Concerning CitE and CitF, this is the first study suggesting PTM regulatory events. The differential analysis showed that anaerobic conditions promote citrate fermentation enzymes accumulation, similarly to what already reported in other enterobacteria [10,30,31]. In addition and more interestingly, during BAS-10 anaerobic growth, Fe(III) positively controls the accumulation of citrate fermentative enzymes better than Na(I). In fact, in *K. oxytoca* BAS-10 citrate fermentation appears much more up-regulated in presence of Fe(III) than Na(I), making this process unique for this microorganism among enterobacteria which usually ferment citrate in a NA(I)-dependent manner [1,8-10].

**Figure 4:**
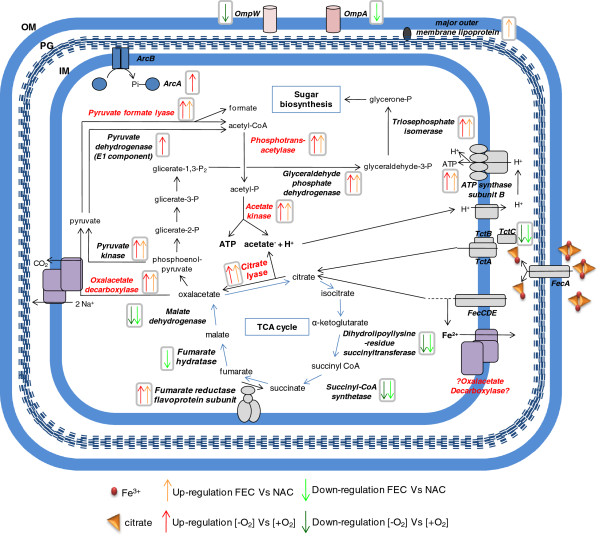
**Synoptic scheme of metabolic pathways involved in Fe(III)-citrate catabolism.** Anabolic routes, regulatory and membrane-associated proteins are also indicated. Reactions are reported according to KEGG [22] and EcoCy [23] databases. OM: outer membrane. PG: peptidoglycan. IM: inner membrane.

Occurrence of Fe(III) during anaerobic growth also modulated the abundance of many central carbon metabolism enzymes. In particular, pyruvate kinase (PK), glyceraldehyde phosphate dehydrogenase (GAPDH) and triosephosphate isomerase (TIM) were up-regulated whereas TCA cycle enzymes, such as dihydrolipoyllysine-residue succinyltransferase (SucB), malate dehydrogenase (MDH) and fumarate hydratase (FH) were down-regulated in anaerobic FEC with respect to both aerobic FEC and anaerobic NAC (Table 1, Figures 2 and 3). Interestingly, fumarate reductase flavoprotein subunit (FrdA) was up-regulated during the anaerobic growth on FEC with respect to both NAC and aerobic FEC (Figure 4). FrdA is part of complex II homolog menaquinol:fumarate oxidoreductase, which oxidizes menaquinol and transfers the electrons to fumarate during bacterial anaerobic respiration, with fumarate as the terminal electron acceptor [32], thus counteracting TCA cycle down-regulation. Altogether these data suggest that to efficiently divert the carbon flux towards acetate and ATP production citrate fermentation enzymes are up-regulated during anaerobic growth on FEC, whereas TCA cycle enzymes are repressed (Table 1 and Figure 4). In addition, these data indicated that the increased catabolism of citrate throughout a fermentative pathway is coupled to the synthesis of metabolic precursors necessary for anabolic processes like sugar biosynthesis. In particular, the TIM product glycerone-P is a precursor involved in rhamnose biosynthesis (Figure 4). Since rhamnose is the major sugar of EPS [17], the observed TIM up-regulation can represent an interesting link between central carbon metabolites and EPS synthesis in *Klebsiella.* As a consequence of the increased anaerobic citrate fermentation, the production of acetic acid may also determine an increment of H^+^ gradient across cell membrane, which positively affects the activity of the ATP synthase complex. In agreement with this view, ATP synthase subunit B was observed as up-regulated under anaerobic conditions in FEC medium, in the respect of both NAC and aerobic FEC (Table 1, Figures 2, 3 and 4).

### Central carbon and nitrogen metabolism key regulators

**Figure 5:**
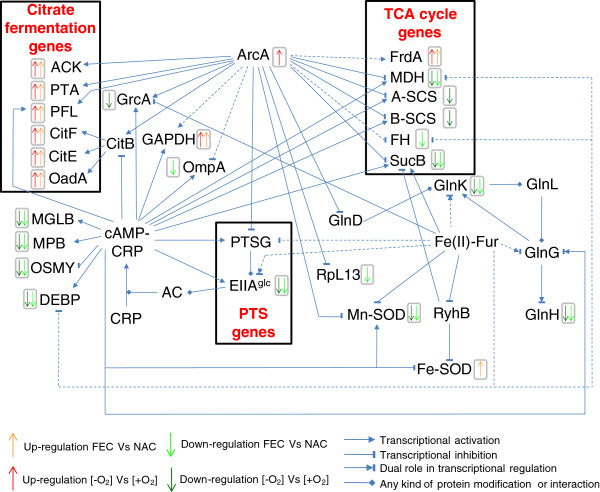
**Regulatory network controlling the expression or the activity of*K. oxytoca*BAS-10 differentially abundant proteins.** The relationships are reported according to EcoCyc database [23], Bott (1997) [1], Salmon et al. (2005) [34] and Kumar et al. (2011) [49]. Dashed lines refer to an indirect relationship.

The proteomic comparison showed down-regulation in anaerobic FEC of EIIA^Glc^, a component of bacterial phosphoenolpyruvate (PEP): carbohydrate phosphotransferase system (PTS), which was revealed as two protein spots differing for Mw in 2D-maps (Table 1, Figures 2 and 3; Additional file 1 Table S1 and Figure S2). The differential regulation of EIIA^Glc^ may be related to the differential regulation of central carbon metabolism enzymes. In fact, in both Gram-negative and Gram-positive bacteria PTS consists of several factors interacting with different regulatory proteins thus controlling glucose metabolism and many other cellular functions [33]. In particular, EIIA^Glc^ is the central processing unit of carbon metabolism in enteric bacteria since it is involved in the regulation of adenylate cyclase (AC) and therefore in carbon catabolite repression trough the control of the catabolite repressor protein (CRP) (Figure 5). In addition, it also interacts with several non-PTS permeases and glycerol kinase to inhibit their activity (inducer exclusion) [33].

Moreover, during anaerobic growth, the down regulation of EIIA^Glc^ may be related to the observed up-regulation of ArcA in FEC with respect to NAC (Table 1; Figures 2 and 3). ArcA, a negative response regulator of genes in aerobic pathways [34,35], is a central regulator of carbon metabolism which, from one hand, negatively controls the expression of *pts* and of TCA cycle genes [33,36] (Figure 5), and, from the other one, it positively regulates the expression of genes, like *citAB*, *pfl* and *ack*, necessary for fermentative metabolic activities under anaerobic conditions [34,37] (Figure 5). In addition to its role of activator of fermentative genes and inhibitor of aerobic catabolism genes ArcA appears to regulate a wide variety of processes like amino acid and sugar transport and metabolism, cofactor and phospholipids biosynthesis, nucleic acid metabolism [34,38].

Interestingly, the observed down-regulation of EIIA^Glc^ in anaerobic FEC with respect to both NAC and aerobic FEC seemed coupled with a differential regulation of important players of nitrogen and amino acid metabolism. In particular, GlnK, identified as three different spots in *K. oxytoca* BAS-10 2D-maps (Table 1, Figure 2 and 3; Additional file 1 Table S1 and Figure S2), is part of nitrogen regulatory system (*ntr*) which facilitates the efficient assimilation of nitrogen from a variety of compounds into glutamine and glutamate as reported in many bacteria like *E. coli*[39]. The activity of GlnK, which is encoded by an operon encoding the ammonia transporter AmtB too, is controlled by uridylylaton state. In nitrogen proficiency, not-uridylylated GlnK form is the most abundant and interacts with and inhibits AmtB [40]. In nitrogen limitation, uridylylated GlnK inhibits the phosphatase activity of the sensor membrane protein GlnL, that forms a two-component system with the regulatory protein GlnG. Phosphorylated GlnG positively affects the expression of many *ntr* genes, including GlnK [39,41] (Figure 5). The difference in pI of the GlnK forms here identified gives count for protein uridylylation, which decreases the predicted pI value of native protein form. The protein form with decreased Mw may be due to the possible removal of a N-terminal amino acid stretch as reported in *Streptomyces coelicolor*[42]. Interestingly, the putative uridylylated GlnK was down-regulated in anaerobic FEC with respect to both anaerobic NAC and aerobic FEC thus suggesting that nitrogen limitation was sensed by BAS-10 growing in anaerobic FEC. The GlnK abundance was in agreement with the down-regulation in anaerobic FEC of carbamoyl-phosphate synthase small subunit (CPSase) in the respect of both NAC and aerobic FEC. In fact, CPSase, an enzymes involved in pyrimidine and arginine biosynthesis, regulates nitrogen disposal by catalyzing synthesis of carbamoyl phosphate from ammonia or glutamine and carbamate [43].

Carbon and nitrogen metabolism could also be associated to the regulation of membrane proteins participating to amino acid up-taking, like the glutamine ABC transporter periplasmic-binding protein (GlnH) and glutamate and aspartate transporter subunit (DEBP), down-regulated in anaerobic FEC with respect to both NAC and aerobic FEC, or arginine 3^rd^ transport system periplasmic-binding protein (ArtJ) and leucine ABC transporter subunit substrate-binding protein (LivK), up-regulated in anaerobic FEC with respect to both NAC and aerobic FEC. In many bacteria, nitrogen and carbon metabolism are linked to cell energetic state through the conversion of TCA cycle intermediate alpha-ketoglutarate and the amino acid glutamate [44-46]. In *K. oxytoca* BAS-10 growing in anaerobic FEC, carbon metabolism regulators, like ArcA and CRP, and nitrogen assimilation factors, such as GlnG, could be coordinately controlled (Figure 5) in order to balance amino acid content and to promote biomass accumulation.

### Redox balance and stress response proteins

Two isoforms of superoxide dismutase Mn-dependent (Mn-SOD) were down-regulated in anaerobic FEC with the respect to both aerobic FEC and anaerobic NAC (Table 1, Figure 2 and 3; Additional file 1 Table S1 and Figure S2). Mn-SOD is devoted to destroy radicals which are normally produced within the cells. Interestingly and in agreement with what already observed in *E. coli*[47] and *S. typhimurium*[48], the presence of iron causes the accumulation of a Fe-SOD whose activity is devoted to contrast iron dependent free radical formation. In particular, Fe-SOD had an abundance level not depending on oxygen, since their relative amount was the same during aerobic and anaerobic growth on FEC. Transcriptional regulation of both SOD genes is reported to be mediated by the Ferric uptake regulator (Fur). Fur controls the transcription of genes, like *fecABCDE*, involved in iron homeostasis and participates also in the transcriptional regulation of many other genes, such as TCA cycle and PTS genes [49] (Figure 5). Indeed, Fur is mainly a transcriptional repressor when it binds Fe(II). Anyway, by repressing the expression of the non-coding RNA Ryhb, that negatively controls mRNA translation into proteins, consequently Fur may also indirectly exert a positive gene regulatory effect as observed in *E. coli*[50]. This phenomenon could also occur in *K. oxytoca* BAS-10 since both *fur* and *ryhb* homologues were identified in *Klebsiella* genus (BLAST analysis, data not shown). Thus, it is likely that the Fe(III)-dependent accumulation of Fe-SOD under anaerobiosis may depend on Fur action to counteract metal toxicity, as observed in *E. coli*[51].

Furthermore, two proteins, the alkyl hydroperoxide reductase subunit C (AHPC) and the thiol peroxidase (TPX), contrasting reactive oxygen species (ROS), were differentially regulated in *K. oxytoca* BAS-10. In particular, AHPC was down-regulated in anaerobic FEC with respect to aerobic FEC, thus revealing an oxygen dependent control, while TPX was down-regulated in anaerobic FEC with respect to anaerobic NAC, thus revealing a Fe(III)-dependent regulation.

Concerning stress associate proteins, the hyperosmotically inducible periplasmic (OSMY) was down-regulated in anaerobic FEC with respect to both aerobic FEC and anaerobic NAC. This protein was found associated with osmotic stress response during *E. coli*[52]. Other stress associated proteins, like DNA protection during starvation protein (DPS) and the universal stress protein F (USF), were down-regulated in anaerobic FEC with the respect to aerobic FEC showing an oxygen dependent regulation. In DPS, a ferritin-like protein, binds to the chromosome and protects DNA from oxidative damage by sequestering intracellular Fe(III) and storing it in the form of Fe(III)-oxyhydroxide mineral [53].

Proteome data revealed that outer membrane proteins are differentially regulated under anaerobic condition in presence of FEC or NAC. In particular, three isoforms of major outer membrane murein-associated lipoprotein (Lpp), differing in pI value, were up-regulated in anaerobic FEC. In *E. coli*, Lpp is synthesized as a precursor protein (prolipoprotein) and processed by a signal peptidase after modification with the addition of di-acyl-glycerol and a fatty acid chain [54]. No modification altering pI has been reported up to day being this study the first one suggesting the possibility of such similar PTMs in Lpp. This finding was coupled with the down-regulation of outer membrane OmpA. Indeed, OmpA was revealed as four down-regulated full size isoforms and one and three protein fragments up- and down-regulated in anaerobic FEC, respectively (Table 1). In *E. coli* OmpA acts as a low permeability porin and it is present in *E. coli* protein 2D-maps as different protein spots suggesting PTM control [55]. Interestingly, both Lpp and OmpA are reported to interact with PAL that is part of Tol-PAL complex, a membrane-spanning multiprotein system that has been reported playing several functions in Gram-negative bacteria, including transport regulation, cell envelope integrity and pathogenicity [56]. In addition, Lpp has been reported to interact with TonB protein, which serves to couple the cytoplasmic membrane proton motive force to the active transport of iron-siderophore complexes, including the Fec system [57]. The differential regulation of Lpp and OmpA suggests differential roles for these two proteins in controlling iron omeostasis, membrane transport and/or preventing possible cellular damage.

In conclusion, this proteomic analysis revealed an iron-dependent regulation for several stress-related proteins; altogether, these observations suggest the occurrence of different concomitant mechanisms to contrast and/or prevent BAS-10 cell damage during aerobic or anaerobic growth.

## Conclusion

At the best of our knowledge, for the first time this study describes at the proteome level biochemical mechanisms allowing the physiological adaptation of the enterobacterium *K. oxytoca strain* BAS-10 [12,16-18] to sustain anaerobic growth on Fe(III)-citrate as sole carbon and energy source. *K. oxytoca* BAS-10, isolated from pyrite mine drainages of Colline Metallifere (Tuscany, Italy) and sharing the same ecological niche of specialized bacteria like *Acidithiobacillus ferrooxidans*[58], has the peculiarity of thriving on high concentrations of Fe(III)-citrate. This character distinguishes it from other clinically-isolated enterobacteria, belonging to *K. oxytoca, K. pneumonia*, *S. typhimurium* and *Neisseria gonorrhoeae* species, which are able to uptake and ferment anaerobically citrate as sole carbon and energy source in a Na(I)-dependent manner only [1,10,12,59]. When anaerobically grown on FEC or NAC, *K. oxytoca* BAS-10 biomass production yields and citrate consumptions were very similar [16]. Under this condition, about half of the initial Fe(III) was reduced to Fe(II) [12,16], the highest levels observed so far with respect to other fermentation processes [60,61].

Differential proteome analyses, carried out using anaerobic FEC as pivotal condition, revealed two sets of proteins whose abundance is O_2_- and Fe(III)-dependent, respectively. The cross checking of these proteome data revealed proteins whose abundance is specifically associated to anaerobic growth on Fe(III). Interestingly, these data portrayed a coordinated regulation of citrate catabolic enzymes during anaerobic growth on FEC. Citrate may enter the cell throughout several kind of permeases, including Cit transporters [1,10], TctABC [3] and FecABCDE systems [4-7]. Then, it is catabolised via the TCA cycle or the citrate fermentation pathway under aerobic or anaerobic growth, respectively. Under anaerobic conditions, the first pathway is repressed, while the second one is favoured. In addition, a fascinating speculation is that one BAS-10 citrate fermentation enzyme, the oxalacetate decarboxylase, can be adapted to act as both Fe(II)- and Na(I)-depending pump to excrete Fe(II) and Na(I). This hypothesis has to be verified by functional studies on BAS-10 *oadGAB* genes. Furthermore, PK, GAPDH and TIM abundance levels suggested an increased synthesis of metabolic precursors necessary for anabolic processes, such as those yielding rhamnose, linking together central carbon metabolism and EPS biosynthesis in *Klebsiella.* Differential regulation of metabolic enzymes was associated with variable cellular levels of carbon metabolism key regulators ArcA and PTS EIIA^Glc^, and important players of nitrogen and amino acid metabolism, like GlnK. In this context, ArcA plays a central role in regulating anaerobic carbon metabolism by controlling the expression of citrate fermentation and TCA cycle enzymes directly or indirectly – i.e. throughout PTS gene regulation – and nitrogen metabolism (Figure 5). Furthermore, during anaerobic FEC growth, ArcA-dependent gene regulation may be combined to iron effect on gene expression trough the activity of Fur (Figure 5). Proteome results also suggest that metabolic pathway modulation seems coupled with regulation of redox balance, stress response and outer membrane protein content. The occurrence of various proteins involved in these processes is finely balanced to face iron and pH toxicity, thus allowing *K. oxytoca* BAS-10 to survive in extreme habitats. Altogether, these data well correlate with previous observations on other microorganisms able surviving in highly polluted environments [62,63] or extremophile organisms living under extreme conditions [64,65], both generally activating multiple intracellular processes devoted to contrast environmental toxic effects and optimize energy production.

According to the results presented here, *K. oxytoca* BAS-10 faces high iron concentration by various molecular adaptation mechanisms, which are summarized in Figures 4 and 5. The coordinated regulation of metabolic pathways and cellular networks allows *K. oxytoca* BAS-10 to efficiently sustain bacterial growth by adapting metabolic and biochemical processes in order to face iron toxicity and to ensure optimization of energy production. In this context, EPS production is intrinsically related to bacterial biomass production and metabolic and energy supply for anabolic reactions. Since *K. oxytoca* BAS-10 was proven to have a high potential for biotechnological applications [18-20], the data presented here could be used for redesigning fermentation strategies that are difficult to be intuitively identified and approaching novel genetic targets to be engineered for a rational design of high-yielding EPS producer strains.

## Methods

### Bacterial cultivation and metal binding exopolysaccharide (EPS) quantification

For *K. oxytoca* BAS-10 cultures, two defined media were prepared containing the same mineral composition (2.5 g NaHCO_3_, 1.5 g NH_4_Cl, 1.5 g MgSO_4_.7H_2_O, 0.6 g NaH_2_PO_4_, 0.1 g KCl, buffered at pH 7.6 with NaOH) and 50 mM Na-citrate (14.7 g.l^-1^), named NAC medium, or 50 mM ferric citrate (13.15 g.l^-1^), named FEC medium, as the sole carbon and energy source. BAS-10 was retrieved from cryovials kept at −80°C in 25% glycerol in Nutrient Broth (NB) (Difco). One ml aliquots of dense (abs_600nm_ = 1.4) overnight NB pre-culture of BAS-10 were used to inoculate (1:100 v:v) FEC or NAC medium cultivations. Aerobic cultivations were performed in flasks, aerated by a magnetic stir bar (250 r.p.m.). Anaerobic cultivations were performed in pirex bottles, previously fluxed with N_2_ and kept under anaerobiosis by sealed cap. Aerobic and anaerobic cultivations were performed in parallel quadruplicates, at 30°C. Biomass samples for proteomic analysis were harvested by centrifugation at late exponential growth stages. i.e. after 36 and 72 h of incubation in aerobic and anaerobic cultivations, respectively. For EPS extraction and quantification, the procedures described by Baldi et al. (2009) [12] were followed.

### Phylogenetic analysis

One microliter of diluted (1:10 in autoclaved distilled water) BAS-10 bacterial suspension was used for PCR amplification of 16S rRNA gene by using fD1 and rD1 universal primers [66] and 1.5 units of recombinant Taq DNA Polymerase (Invitrogen, Life Technologies), according to manufacturer’s specifications. To perform bacterial cell lysis and DNA denaturation, a treatment of 5 min, at 95 °C, was performed. Then, amplification steps were carried out for 40 cycles consisting of 1 min at 95°C, 1 min at 55°C and 2 min at 72 °C, with a final extension of 10 min at 72 °C. PCR fragments were purified by using PCR clean-up kit (NucleoSpin, Macherey-Nagel) according to manufacturer’s specifications and sequenced with the same primers at BMR Genomics (University of Padova, Italy).

BAS-10 16S rDNA sequence was compared using BLAST probing [67] of DNA sequences from the NCBI database (http://blast.ncbi.nlm.nih.gov/Blast.cgi?CMD=Web&PAGE_TYPE=BlastHome) with default parameters, selecting either only cultivable reference strains (refseq_rna) or whole database strains (rn/nt). ClustalX program [68] was used to align the homologous 16S rDNA gene sequences obtained from the database, choosing the slow/accurate method for pairwise alignment. All positions containing alignment gaps were eliminated in pairwise sequence comparisons by selecting the related option. The 16S rDNA sequence of *Streptomyces tendae* was used as outgroup. Alienated sequences were then used to generate rooted trees throughout Unweighted Pair Group Method with Arithmetic Mean (UPGMA) algorithm.

### Protein extraction

Total proteins were extracted from frozen biomass samples of quadruplicated cultivations *per* each condition by using an experimental procedure previously described [69,70]. Briefly, pellets were cleaned three times with a washing solution (10 mM Tris–HCl pH 7.5, 5 mM EDTA, 1 mM DTT, 0.5 mM PMSF, 4 mg/ml leucopeptin, 0.7 mg/ml pepstatin, 5 mg/ml benzamidin). Following re-suspension in washing solution containing 0.3% SDS, bacterial cells were disrupted by sonication on ice (output control 4, 4 x 15 s, Vibra Cell, USA). Samples were then boiled (5 min) and rapidly cooled down on ice (15 min). DNase (100 μg/ml) and RNase (50 μg/ml) were added in ice for 20 min. Cell debris and non-broken cells were separated by centrifugation at 15,000 x *g*, for 15 min, at 4°C. Protein solutions were dialyzed against distilled water for 3 h, at 4°C, precipitated by using 3 vol of acetone, at −20°C, overnight, and re-suspended in 30 mM Tris pH 8.5, 7 M urea, 2 M thiourea, 4% w/v CHAPS.

### 2D-DIGE analysis

Proteomes of *K. oxytoca* BAS-10 grown in FEC medium under anaerobiosis [−O_2_ and aerobiosis [+O_2_ or in NAC under [−O_2_ conditions were compared, using FEC medium under [−O_2_ as pivotal condition. To this aim, protein samples were labelled for 2D-DIGE analysis using CyDye^TM^ DIGE minimal labelling kit (GE Healthcare, Sweden), as previously described [71]. Briefly, protein samples (40 μg) were labelled with 320 pmol of CyDye on ice in the dark for 30 min. Labelling reaction was stopped by addition of 0.8 μl of 10 mM lysine and incubation was continued on ice for 15 min., in the dark. Two samples out of a total of four *per* each condition were labelled using Cy3 dye and the other two using Cy5 dye to account for florescence bias. In addition, a standard protein pool was generated by combining an equal amount of protein extracts from which six 40 μg protein aliquots were then minimally labelled with 320 pmol Cy2 fluorescent dye (CyDye^TM^ DIGE, GE Healthcare), according to manufacturer’s instructions. Thus, a total of six 2D-DIGE gels were performed containing a mix of Cy2-labeled pooled protein standard and Cy3- and Cy5-labeled protein samples from cells incubated in FEC medium under [−O_2_ and [+O_2_ and in NAC under [−O_2_ conditions, analysed in couples, i.e. FEC [−O_2_*vs* FEC [+O_2_, FEC [−O_2_*vs* NAC [−O_2_ and FEC [+O_2_*vs* NAC [−O_2_. For IEF, DeStreak rehydration solution (GE Healthcare), containing 0.68% v/v IPG buffer (GE Healthcare) and 10 mM DTT (Sigma), was added to each mix up to a 340 μl final volume. IEF was performed as previously described [57] using 4–7 pH range 18 cm-IPG strips (GE Healthcare) in an Ettan IPGphor III apparatus (GE Healthcare). After IEF, IPG strips were incubated with an equilibration buffer (6 M urea, 30% v/v glycerol, 2% w/v SDS, 0.05 M Tris–HCl, pH 6.8) containing 2% w/v DTE for 10 min; thiol groups were then blocked by further incubation with equilibration buffer containing 2.5% w/v iodoacetamide. Focused proteins were then separated by using 12% SDS-PAGE, at 10°C, in a Hettan Dalt six (GE Healthcare), with a maximum setting of 40 μA *per* gel and 110 V.

### Protein visualization and image analysis

The 2D-gels were scanned with a DIGE imager (GE Healthcare) to detect cyanin-labeled proteins, according to manufacturer’s instructions. Differential gel analysis was performed automatically by using the Image Master 2D Platinum 7.0 DIGE software (GE Healthcare), according to the manufacturer’s instructions. Protein spots were automatically detected and then matched. Individual spot abundance was automatically calculated from quadruplicated 2D-gels as mean spot volume, i.e. integration of optical density over spot area, and normalized to the Cy2-labeled internal pooled protein standard. Protein spots showing more than 1.5 fold change in spot volume (increased for up-regulation or decreased for down-regulation), with a statistically significant ANOVA value (*P* ≤ 0.05), were considered differentially represented and further identified by MS analysis.

### Protein identification

Protein spots were excised from the 2D-gels, alkylated, digested with trypsin and identified as previously reported [56]. Peptide mixtures were desalted by μZip-TipC18 (Millipore, MA) using 50% v/v acetonitrile/5% v/v formic acid as eluent before MALDI-TOF-MS and nLC-ESI-LIT-MS/MS analysis.

In the case of MALDI-TOF-MS experiments, peptide mixtures were loaded on the MALDI target, by using the dried droplet technique, α-cyano-4-hydroxycinnamic acid as matrix, and analyzed using a Voyager-DE PRO mass spectrometer (Applied Biosystems, USA) operating in positive ion reflectron, with an acceleration voltage of 20 kV, a N_2_ laser (337 nm) and a laser repetition rate of 4 Hz [72]. Final mass spectra, measured over a mass range of 700–4000 Da and obtained by averaging 400–800 laser shots, were elaborated using the DataExplorer 5.1 software (Applied Biosystems) and manually inspected to get the corresponding peak lists. Internal mass calibration was performed with peptides deriving from trypsin autoproteolysis. Tryptic digests were eventually analyzed by nLC-ESI-LIT-MS/MS using a LTQ XL mass spectrometer (Thermo, San Jose, CA) equipped with a Proxeon nanospray source connected to an Easy-nanoLC (Proxeon, Odense, Denmark) [73,74]. Peptide mixtures were separated on an Easy C18 column (10–0.075 mm, 3 μm) (Proxeon). Mobile phases were 0.1% v/v aqueous formic acid (solvent A) and 0.1% v/v formic acid in acetonitrile (solvent B), running at total flow rate of 300 nL/min. Linear gradient was initiated 20 min after sample loading; solvent B ramped from 5% to 35% over 15 min, from 35% to 95% over 2 min. Spectra were acquired in the range m/z 400–1800. Acquisition was controlled by a data-dependent product ion scanning procedure over the 3 most abundant ions, enabling dynamic exclusion (repeat count 2 and exclusion duration 60 s); the mass isolation window and collision energy were set to *m/z* 3 and 35%, respectively.

MASCOT search engine version 2.2.06 (Matrix Science, UK) was used to identify protein spots from an updated NCBI non-redundant database by using MALDI-TOF-MS and nLC-ESI-LIT-MS/MS data and by selecting trypsin as proteolytic enzyme, a missed cleavages maximum value of 2, Cys carbamidomethylation and Met oxidation as fixed and variable modification, respectively. In the first case, a mass tolerance value of 40–80 ppm was selected; in the second case, a mass tolerance value of 2 Da for precursor ion and 0.8 Da for MS/MS fragments was chosen. MALDI-TOF-PMF candidates with a MASCOT score > 83 or nLC-ESI-LIT-MS/MS candidates with more than 2 assigned peptides with an individual MASCOT score > 25, both corresponding to *P* < 0.05 for a significant identification, were further evaluated by the comparison with their calculated mass and pI values, using the experimental data obtained from 2D-DIGE.

## Abbreviations

2D-DIGE: 2D-Differential Gel Electrophoresis; MS: Mass Spectrometry; FEC: Fe(III)-Citrate; NAC: Na(I)-Citrate; EPS: Exopolysaccharide; PTM(s): Post-Translational Modification(s); AC: Adenylate Cyclase; CRP: Catabolite Repressor Protein.

## Competing interests

The authors declare that they have no competing interests.

## Authors’ contribution

GG carried out 16S rDNA amplification, phylogenetic analysis, 2D-DIGE analysis, gene ontology and wrote the draft manuscript. FB designed and performed BAS10 cultivations and helped to wrote the draft manuscript. GR carried out protein MS-identification. MG helped to performe BAS10 cultivations. AC helped to perform 16S rDNA amplification, phylogenetic analysis and 2D-DIGE experiments. AS supervised protein MS-identification and revised the manuscript. AMP conceived and supervised the study and participated in its design and coordination and revised the manuscript. All authors read and approved the final manuscript.

## Supplementary Material

Additional file 1 **Table S1:containing mass spectrometry parameters of *K. oxytoca *BAS-10 differentially abundant protein identification;Figure S1:showing phylogenetic tree generated by using 16S rDNA sequence of*K. oxytoca*BAS-10 and the first twenty hits from a BLAST analysis performed by selecting whole database strains; Figure S2 showing 2D-protein maps, chosen as examples of anaerobic FEC, anaerobic NAC and aerobic FEC condition, respectively.** Labels indicating differentially abundant protein spots and referring to Table 1 and Table 1 are also reported in **Figure S2**; **Figure S3:** containing distribution into functional classes of the identified protein spots, according to KEGG metabolic database (http://www.genome.jp/kegg/), from the comparison between anaerobic and aerobic growth on FEC from the comparison between anaerobic growth on FEC and NAC, respectively; 1447 nt gene sequence generated from BAS-10 16S rDNA. (DOC 1711 kb) Click here for file
