# Supplementary material for: Adaptative biochemical pathways and regulatory networks in Klebsiella oxytoca BAS-10 producing a biotechnologically relevant exopolysaccharide during Fe(III)-citrate fermentation
Source: Microb Cell Fact. 2012 Nov 23;11:152. doi: 10.1186/1475-2859-11-152 (PMC3539929; doi:10.1186/1475-2859-11-152)
Supplement: Additional file 1 — Table S1:containing mass spectrometry parameters of K. oxytoca BAS-10 differentially abundant protein identification;Figure S1:showing phylogenetic tree generated by using 16S rDNA sequence ofK. oxytocaBAS-10 and the first twenty hits from a BLAST analysis performed by selecting whole database strains; Figure S2 showing 2D-protein maps, chosen as examples of anaerobic FEC, anaerobic NAC and aerobic FEC condition, respectively. Labels indicating differentially abundant protein spots and referring to Table 1 and Table 1 are also reported in Figure S2; Figure S3: containing distribution into functional classes of the identified protein spots, according to KEGG metabolic database (http://www.genome.jp/kegg/), from the comparison between anaerobic and aerobic growth on FEC from the comparison between anaerobic growth on FEC and NAC, respectively; 1447 nt gene sequence generated from BAS-10 16S rDNA. (DOC 1711 kb) [file 1475-2859-11-152-S1.doc]

**Adaptative biochemical pathways and regulatory networks in *Klebsiella oxytoca* BAS-10 producing a biotechnologically relevant exopolysaccharide during Fe(III)-citrate fermentation**

**Giuseppe Gallo^1*^, Franco Baldi^2*^, Gianni Renzone^3^, Michele Gallo^2^, Antonio Cordaro^1^, Andrea Scaloni^3^ and Anna Maria Puglia^1^**

^a^Dipartimento di Scienze e Tecnologie Molecolari e Biomolecolari (STEMBIO), Università di Palermo, 90128 Palermo, Italy

^b^Dipartimento di Scienze Molecolari e Nanosistemi (DSMN), Cà Foscari, Università di Venezia, 30123 Venezia, Italy

^c^Laboratorio di Proteomica e Spettrometria di Massa, ISPAAM, Consiglio Nazionale delle Ricerche, 80147 Napoli, Italy

**ADDITIONAL FILE 1**

**Table 1S. Mass spectrometry-based identification of *K. oxytoca* BAS-10 protein spots differentially regulated during anaerobic growth in FEC.**

| **Spot** | **Protein name** | **Acronym** | **NCBI code** | **Theor.**  **pI/Mr (kDa)** | **Exp. pI/Mr (kDa)** | **ID method ^a^** | **Matched peptides** | **Sequence coverage** | **Mascot Score** | **Matched/**  **searched**  **peptides** | **Organism** |
| --- | --- | --- | --- | --- | --- | --- | --- | --- | --- | --- | --- |
| 1 | NADP-specific glutamate dehydrogenase | GLDH | 376386651 | 6.73/49 | 6.26/45 | TMS | 10 | 28 | 609 |  | *K. oxytoca 10-5243* |
| 2 | Fumarate reductase flavoprotein subunit | FrdA | 376396036 | 5.60/66 | 5.44/77 | TMS | 16 | 34 | 960 |  | *K. oxytoca 10-5250* |
| 3 | Ketol-acid reductoisomerase | IlvC | 376396684 | 5.25/54 | 5.04/54 | TMS | 27 | 54 | 1370 |  | *K. oxytoca 10-5250* |
| 4 | D-3-phosphoglycerate dehydrogenase | PHGDH | 365906793 | 6.06/44 | 5.92/46 | TMS | 6 | 18 | 279 |  | *K. oxytoca KCTC 1686* |
| 5 | Nitrogen regulatory protein P-II 2 | GlnK | 376394829 | 5.84/12 | 5.62/13 | PMF | 10 | 73 | 181 | 10/12 | *K. oxytoca 10-5250* |
| 6 | Nitrogen regulatory protein P-II 2 | GlnK | 376394829 | 5.84/12 | 5.62/13 | TMS | 3 | 42 | 313 |  | *K. oxytoca 10-5250* |
| 7 | Nitrogen regulatory protein P-II 2 | GlnK | 376394829 | 5.84/12 | 5.20/13 | TMS | 4 | 50 | 255 |  | *K. oxytoca 10-5250* |
| 8 | Major outer membrane lipoprotein 1 | Lpp | 376401994 | 9.36/8 | 4.16/8 | TMS | 2 | 33 | 133 |  | *K. oxytoca 10-5250* |
| 9 | Major outer membrane lipoprotein 1 | Lpp | 376401995 | 9.36/9 | 4.04/12 | TMS | 3 | 33 | 144 |  | *K. oxytoca 10-5250* |
| 10 | Major outer membrane lipoprotein 1 | Lpp | 376401994 | 9.36/8 | 4.44/12 | TMS | 3 | 33 | 156 |  | *K. oxytoca 10-5250* |
| 11 | Thiol peroxidase | TPX | 376401245 | 4.67/18 | 4.8/19 | TMS | 3 | 25 | 188 |  | *K. oxytoca 10-5250* |
| 12 | Acetate kinase A/propionate kinase 2 | ACK | 365911543 | 5.84/44 | 5.39/27 | TMS | 8 | 24 | 364 |  | *K. oxytoca KCTC 1686* |
| 13 | Outer membrane protein A | OmpA | 376393752 | 5.98/38 | 4.98/31 | TMS | 12 | 42 | 776 |  | *K. oxytoca 10-5250* |
| 14 | Citrate lyase subunit beta | CitE | 376394377 | 5.43/31 | 4.87/29 | TMS | 11 | 46 | 652 |  | *K. oxytoca 10-5250* |
| 15 | Citrate lyase, alpha subunit | CitF | 365908401 | 5.94/55 | 5.98/55 | TMS | 13 | 27 | 792 |  | *K. oxytoca KCTC 1686* |
| 16 | Citrate lyase, alpha subunit | CitF | 365908401 | 5.94/55 | 5.89/55 | TMS | 13 | 31 | 757 |  | *K. oxytoca KCTC 1686* |
| 17 | Hypothetical protein HMPREF9694_04828 (dihydrolipoyllysine-residue succinyltransferase, E2 component) | SucB | 376395176 | 5.74/44 | 5.39/54 | TMS | 18 | 46 | 1048 |  | *K. oxytoca 10-5250* |
| 18 | Hypothetical protein HMPREF9694_01670 (tricarboxylic transport) | TctC | 376400424 | 8.61/35 | 6.7/26 | TMS | 7 | 30 | 408 |  | *K. oxytoca 10-5250* |
| 19 | Glucose-specific phosphotransferase enzyme IIA component | EIIA^Glc^ | 376399349 | 4.73/18 | 4.6/22 | TMS | 3 | 21 | 129 |  | *K. oxytoca 10-5250* |
| 20 | Glucose-specific phosphotransferase enzyme IIA component | EIIA^Glc^ | 376399349 | 4.73/18 | 4.62/20 | TMS | 3 | 24 | 171 |  | *K. oxytoca 10-5250* |
| 21 | Glyceraldehyde-3-phosphate dehydrogenase | GAPDH | 376401359 | 6.33/36 | 6.2/31 | TMS | 3 | 14 | 120 |  | *K. oxytoca 10-5250* |
| 22 | Malate dehydrogenase | MDH | 376398117 | 5.57/33 | 5.34/34 | TMS | 10 | 48 | 513 |  | *K. oxytoca 10-5250* |
| 23 | Malate dehydrogenase | MDH | 376398117 | 5.57/33 | 5.08/29 | TMS | 8 | 38 | 437 |  | *K. oxytoca 10-5250* |
| 24 | Oxaloacetate decarboxylase alpha chain | OadA | 376376739 | 5.44/63 | 5.04/64 | TMS | 12 | 18 | 519 |  | *K. oxytoca 10-5243* |
| 25 | Oxaloacetate decarboxylase alpha chain | OadA | 376376739 | 5.44/63 | 4.7/43 | TMS | 8 | 18 | 517 |  | *K. oxytoca 10-5243* |
| 26 | Citrate lyase subunit beta | CitE | 376376734 | 5.35/32 | 5.07/32 | TMS | 4 | 19 | 199 |  | *K. oxytoca 10-5243* |
| 27 | Phosphotrans-acetylase | PTA | 365911544 | 5.26/77 | 5.27/65 | TMS | 9 | 17 | 492 |  | *K. oxytoca KCTC 1686* |
| 28 | Pyruvate dehydrogenase subunit E1 | PDH | 365908482 | 5.47/100 | 5.52/67 | TMS | 12 | 15 | 737 |  | *K. oxytoca KCTC 1686* |
| 29 | Pyruvate formate lyase | PFL | 376393708 | 5.63/85 | 5.09/29 | TMS | 6 | 11 | 284 |  | *K. oxytoca 10-5250* |
| 30 | Pyruvate formate lyase | PFL | 376393708 | 5.63/85 | 5.3/78 | PMF | 12 | 15 | 156 | 12/12 | *K. oxytoca 10-5250* |
| 31 | Pyruvate formate lyase | PFL | 376393708 | 5.63/85 | 5.37/78 | PMF | 19 | 24 | 233 | 19/21 | *K. oxytoca 10-5250* |
| 32 | Pyruvate formate lyase | PFL | 376393708 | 5.63/85 | 5.44/77 | PMF | 17 | 20 | 201 | 17/19 | *K. oxytoca 10-5250* |
| 33 | Succinyl-CoA synthetase subunit beta | B-SCS | 365909191 | 5.35/42 | 5.05/43 | TMS | 6 | 17 | 343 |  | *K. oxytoca KCTC 1686* |
| 34 | Leucine ABC transporter subunit substrate-binding protein LivK | LivK | 365907255 | 5.71/40 | 5.03/42 | TMS | 7 | 24 | 420 |  | *K. oxytoca KCTC 1686* |
| 35 | Pyruvate formate lyase | PFL | 376393708 | 5.63/85 | 5.16/43 | TMS | 5 | 8 | 282 |  | *K. oxytoca 10-5250* |
| 36 | Citrate lyase subunit beta | CitE | 376376734 | 5.35/32 | 5.19/31 | TMS | 6 | 27 | 307 |  | *K. oxytoca 10-5243* |
| 37 | Aerobic respiration control protein ArcA | ARCA | 376394332 | 5.30/27 | 5.04/29 | TMS | 6 | 34 | 373 |  | *K. oxytoca 10-5250* |
| 38 | Pyruvate kinase | PK | 365911037 | 6.00/52 | 5.78/55 | TMS | 15 | 43 | 783 |  | *K. oxytoca KCTC 1686* |
| 39 | Outer membrane protein A | OmpA | 376393752 | 5.98/38 | 5.5/28 | TMS | 13 | 48 | 738 |  | *K. oxytoca 10-5250* |
| 40 | 3-methyl-2-Oxobutanoate hydroxymethyltransferase | PanB | 365908516 | 5.64/28 | 5.34/29 | TMS | 2 | 9 | 134 |  | *K. pneumoniae 342* |
| 41 | Succinyl-CoA synthetase subunit alpha | A-SCS | 365909192 | 5.89/30 | 5.65/30 | TMS | 10 | 25 | 549 |  | *K. oxytoca KCTC 1686* |
| 42 | Fumarate hydratase class II | FH | 376401593 | 6.02/50 | 5.92/46 | TMS | 9 | 20 | 395 |  | *K. oxytoca 10-5250* |
| 43 | Outer membrane protein A | OmpA | 376393752 | 5.98/38 | 4.78/32 | TMS | 10 | 45 | 754 |  | *K. oxytoca 10-5250* |
| 44 | Outer membrane protein A | OmpA | 376393752 | 5.98/38 | 4.86/33 | TMS | 14 | 61 | 860 |  | *K. oxytoca 10-5250* |
| 45 | Outer membrane protein W | OmpW | 365909926 | 6.17/23 | 5.2/22 | TMS | 6 | 44 | 447 |  | *K. oxytoca KCTC 1686* |
| 46 | Triosephosphate isomerase | TIM | 365911360 | 5.82/26 | 5.5/26 | TMS | 2 | 12 | 137 |  | *K. oxytoca KCTC 1686* |
| 47 | Universal stress protein F | USF | 376401205 | 5.46/16 |  | TMS | 5 | 25 | 161 |  | *K. oxytoca 10-5250* |
| 48 | Hypothetical protein HMPREF9694_01670 (tricarboxylic transport) | TctC | 376400424 | 8.61/35 | 7.8/29 | TMS | 7 | 24 | 345 |  | *K. oxytoca 10-5250* |
| 49 | 6,7-Dimethyl-8-ribityllumazine synthase | RibH | 365908789 | 5.12/16 | 4.87/14 | TMS | 8 | 62 | 630 |  | *K. oxytoca KCTC 1686* |
| 50 | DNA-binding protein HU-alpha | HU-2 | 376396790 | 9.40/9 | 9.07/11 | TMS | 4 | 34 | 257 |  | *K. oxytoca 10-5250* |
| 51 | DNA protection during starvation protein | DPS | 376393609 | 5.72/19 | 5.61/16 | PMF | 18 | 87 | 276 | 18/19 | *K. oxytoca 10-5250* |
| 52 | ATP synthase subunit beta | ATPβ | 376397178 | 4.93/50 | 4.81/51 | PMF | 10 | 25 | 138 | 10/14 | *K. oxytoca 10-5250* |
| 53 | ABC transporter arginine-binding protein 1 | ArtJ | 376393664 | 6.90/27 | 5.98/25 | TMS | 16 | 62 | 828 |  | *K. oxytoca 10-5250* |
| 56 | D-galactose-binding periplasmic protein | MGLB | 376381367 | 6.14/36 | 5.42/29 | TMS | 6 | 24 | 391 |  | *K. oxytoca 10-5245* |
| 57 | Glutamate and aspartate transporter subunit | DEBP | 365909131 | 8.61/33 | 7.85/30 | TMS | 8 | 33 | 521 |  | *K. oxytoca KCTC 1686* |
| 58 | Glutamine-binding periplasmic protein | GnlH | 376388520 | 8.74/27 | 5.81/26 | TMS | 6 | 32 | 379 |  | *K. oxytoca 10-5243* |
| 59 | Glutamine-binding periplasmic protein | GnlH | 376388520 | 8.74/27 | 7.8/25 | TMS | 4 | 23 | 308 |  | *K. oxytoca 10-5243* |
| 60 | Glutamine-binding periplasmic protein | GnlH | 376388520 | 8.74/27 | 6.87/25 | TMS | 5 | 29 | 407 |  | *K. oxytoca 10-5243* |
| 61 | Maltose ABC transporter periplasmic protein | MBP | 365907919 | 6.88/43 | 5.96/40 | TMS | 11 | 26 | 593 |  | *K. oxytoca KCTC 1686* |
| 62 | Outer membrane protein A | OmpA | 376393752 | 5.98/38 | 5.17/32 | PMF | 21 | 64 | 320 | 21/26 | *K. oxytoca 10-5250* |
| 63 | Outer membrane protein A | OmpA | 376393752 | 5.98/38 | 5.03/32 | PMF | 10 | 34 | 144 | 10/16 | *K. oxytoca 10-5250* |
| 64 | Outer membrane protein A | OmpA | 376393752 | 5.98/38 | 5.39/22 | TMS | 5 | 18 | 328 |  | *K. oxytoca 10-5250* |
| 65 | Carbamoyl phosphate synthase small subunit | CPSase | 365908404 | 5.79/42 | 5.4/42 | TMS | 4 | 16 | 297 |  | *K. oxytoca KCTC 1686* |
| 66 | Multifunctional nucleoside diphosphate kinase | NdK | 365911711 | 5.55/15 | 5.38/14 | TMS | 4 | 43 | 268 |  | *K. oxytoca KCTC 1686* |
| 67 | Nucleoside diphosphate kinase | NdKs | 376399440 | 5.55/15 | 5.42/14 | TMS | 4 | 40 | 197 |  | *K. oxytoca 10-5250* |
| 68 | Adenosine-3'(2'),5'-bisphosphate nucleotidase | CysQ | 365908080 | 5.67/28 | 5.31/29 | TMS | 7 | 30 | 266 |  | *K. oxytoca KCTC 1686* |
| 69 | Osmotically-inducible protein Y | OSMY | 376376350 | 8.67/21 | 5.84/22 | TMS | 6 | 48 | 441 |  | *K. oxytoca 10-5243* |
| 71 | Hypothetical protein HMPREF9694_01670 (tricarboxylic transport) | TctC | 376400424 | 8.61/35 | 8.37/29 | TMS | 15 | 55 | 765 |  | *K. oxytoca 10-5250* |
| 72 | Alkyl hydroperoxide reductase subunit C | AHPC | 376395087 | 5.03/21 | 4.95/23 | TMS | 7 | 49 | 470 |  | *K. oxytoca 10-5250* |
| 73 | Superoxide dismutase [Mn] | MnSOD | 376396893 | 6.23/23 | 5.84/25 | TMS | 2 | 14 | 193 |  | *K. oxytoca 10-5250* |
| 74 | Superoxide dismutase [Mn] | MnSOD | 376396893 | 6.23/23 | 6.01/24 | TMS | 9 | 42 | 484 |  | *K. oxytoca 10-5250* |
| 75 | Superoxide dismutase [Mn] | MnSOD | 376396893 | 6.23/23 | 5.62/24 | TMS | 4 | 18 | 232 |  | *K. oxytoca 10-5250* |
| 76 | Superoxide dismutase [Fe] | FeSOD | 376402095 | 5.75/21 | 5.49/22 | TMS | 7 | 58 | 326 |  | *K. pneumoniae subsp. pneumoniae MGH 78578* |
| 78 | Autonomous glycyl radical cofactor GrcA | GrcA | 365911773 | 4.82/14 | 4.71/12 | TMS | 3 | 31 | 176 |  | *K. oxytoca KCTC 1686* |
| 79 | Chaperonin | CHA10 | 376396016 | 5.38/10 | 4.97/16 | TMS | 3 | 42 | 180 |  | *K. oxytoca 10-5250* |
| 80 | Ribosomal protein L13 | RPL13 | 365907069 | 9.60/16 | 8.84/16 | TMS | 6 | 57 | 277 |  | *K. pneumoniae subsp. pneumoniae MGH 78578* |
| 81 | Oxaloacetate decarboxylase alpha chain | OadA | 376376739 | 5.44/63 | 4.74/43 | TMS | 6 | 15 | 356 |  | *K. oxytoca 10-5243* |
| 82 | Elongation factor G | EF-G | 365907166 | 5.17/77 | 5.62/50 | TMS | 4 | 8 | 251 |  | *K. oxytoca KCTC 1686* |
| 85 | Peptidyl-prolyl cis-trans isomerase SurA | SurA | 365908414 | 6.42/47 | 5.68/48 | TMS | 9 | 20 | 563 |  | *K. oxytoca KCTC 1686* |
| 86 | Ribosomal protein L1 | RPL1 | 376396770 | 9.56/25 | 7.77/27 | TMS | 2 | 13 | 167 |  | *K. oxytoca 10-5250* |
| 87 | Ribosomal protein L1 | RPL1 | 206564980 | 9.56/25 | 7.56/28 | TMS | 6 | 32 | 293 |  | *K. pneumoniae 342* |
| 88 | Outer membrane protein A | OmpA | 376393752 | 5.98/38 | 5.46/27 | PMF | 8 | 27 | 128 | 8/10 | *K. oxytoca 10-5250* |
| 89 | Isochorismatase hydrolase |  | 365909686 | 5.61/24 | 5.36/26 | TMS | 5 | 34 | 331 |  | *K. oxytoca KCTC 1686* |

^a^ Peptide mass fingerprinting, PMF; nanoLC-ESI-LIT-MS/MS, TMS.

^b^ Relative fold change was measured according to the criteria reported in the experimental section. FEC: Fe(III)-citrate containing medium. NAC: Na(I)-citrate containing medium. +O2: aerobiosis. -O2: anaerobiosis.

***K. oxytoca* BAS 10 16S rDNA sequence:**

CTACCATGCAAGTCGAACGGTAGCACAGAGAGCTTGCTCTCGGGTGACGAGTGGCGGACGGGTGAGTAATGTCTGGGAAACTGCCTGATGGAGGGGGATAACTACTGGAAACGGTAGCTAATACCGCATAACGTCGCAAGACCAAAGAGGGGGACCTTCGGGCCTCTTGCCATCAGATGTGCCCAGATGGGATTAGCTAGTAGGTGGGGTAACGGCTCACCTAGGCGACGATCCCTAGCTGGTCTGAGAGGATGACCAGCCACACTGGAACTGAGACACGGTCCAGACTCCTACGGGAGGCAGCAGTGGGGAATATTGCACAATGGGCGCAAGCCTGATGCAGCCATGCCGCGTGTATGAAGAAGGCCTTCGGGTTGTAAAGTACTTTCAGCGGGGAGGAAGGGAGTAAGGTTAATAACCTTGTTCATTGACGTTACCCGCAGAAGAAGCACCGGCTAACTCCGTGCCAGCAGCCGCGGTAATACGGAGGGTGCAAGCGTTAATCGGAATTACTGGGCGTAAAGCGCACGCAGGCGGTCTGTCAAGTCGGATGTGAAATCCCCGGGCTCAACCTGGGAACTGCATTCGAAACTGGCAGGCTGGAGTCTTGTAGAGGGGGGTAGAATTCCAGGTGTAGCGGTGAAATGCGTAGAGATCTGGAGGAATACCGGTGGCGAAGGCGGCCCCCTGGACAAAGACTGACGCTCAGGTGCGAAAGCGTGGGGAGCAAACAGGATTAGATACCCTGGTAGTCCACGCTGTAAACGATGTCGACTTGGAGGTTGTTCCCTTGAGGAGTGGCTTCCGGAGCTAACGCGTTAAGTCGACCGCCTGGGGAGTACGGCCGCAAGGTTAAAACTCAAATGAATTGACGGGGGCCCGCACAAGCGGTGGAGCATGTGGTTTAATTCGATGCAACGCGAAGAACCTTACCTACTCTTGACATCCAGAGAACTTAGCAGAGATGCTTTGGTGCCTTCGGGAACTCTGAGACAGGTGCTGCATGGCTGTCGTCAGCTCGTGTTGTGAAATGTTGGGTTAAGTCCCGCAACGAGCGCAACCCTTATCCTTTGTTGCCAGCGGTCCGGCCGGGAACTCAAAGGAGACTGCCAGTGATAAACTGGAGGAAGGTGGGGATGACGTCAAGTCATCATGGCCCTTACGAGTAGGGCTACACACGTGCTACAATGGCATATACAAAGAGAAGCGACCTCGCGAGAGCAAGCGGACCTCATAAAGTATGTCGTAGTCCGGATTGGAGTCTGCAACTCGACTCCATGAAGTCGGAATCGCTAGTAATCGTGGATCAGAATGCCACGGTGAATACGTTCCCGGGCCTTGTACACACCGCCCGTCACACCATGGGAGTGGGTTGCAAAAGAAGTAGGTAGCTTAACCTTCGGGAGGGCGCTTACCA CTTTGTGATTCATGACTGGGGTGAAGTCGA

**Figure 1S.** Phylogenetic analysis of BAS-10 strain performed by using 16S rDNA sequences. The first twenty hits from BLAST analysis performed by selecting whole database strains. The 16S rDNA sequence of *Streptomyces tendae* was used as outgroup. Distance unit is based on sequence identity. NCBI accession number of each 16S rDNA is reported after hyphen.


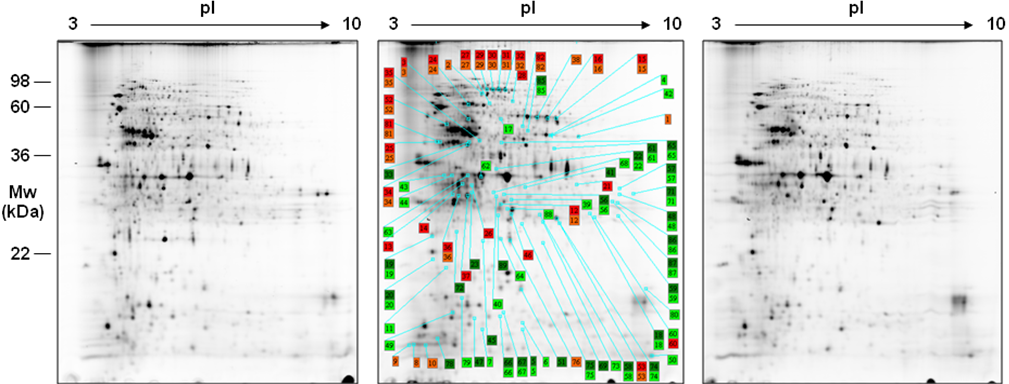


**Figure 2S.** Representative 2D-DIGE maps of *K. oxytoca* BAS10 proteins from aerobic FEC (A), anaerobic FEC (B) and anaerobic NAC (C) cultivations. Labels indicate differentially abundant protein spots identified by MS procedures (Tab. 1 and Tab. 1S). Red and dark-green labels: up- and down-regulation in anaerobic FEC in respect to aerobic FEC, respectively. Orange and light-green labels: up- and down-regulation in anaerobic FEC in respect to anaerobic NAC.

**Figure 3S.** Distribution into functional classes of the identified protein spots according to KEGG metabolic database (http://www.genome.jp/kegg/). A) Differentially represented protein spots resulting from the comparison between anaerobic and aerobic growth on FEC. B) Differentially represented protein spots resulting from the comparison between anaerobic growth on FEC and NAC.
